# Influence of Incubation Temperature and Relative Humidity on the Egg Hatchability Pattern of Two-Spotted (*Gryllus bimaculatus*) and House (*Acheta domesticus*) Crickets

**DOI:** 10.3390/ani14152176

**Published:** 2024-07-26

**Authors:** Jamlong Mitchaothai, Rachakris Lertpatarakomol, Tassanee Trairatapiwan, Achara Lukkananukool

**Affiliations:** 1 Office of Administrative Interdisciplinary Program on Agricultural Technology, School of Agricultural Technology, King Mongkut’s Institute of Technology Ladkrabang (KMITL), Bangkok 10520, Thailand; 2 Faculty of Veterinary Medicine, Mahanakorn University of Technology (MUT), Bangkok 10530, Thailand; rachakris@gmail.com (R.L.); tassanee@mut.ac.th (T.T.); 3 Department of Animal Production Technology and Fisheries, School of Agricultural Technology, King Mongkut’s Institute of Technology Ladkrabang (KMITL), Bangkok 10520, Thailand; achara.lu@kmitl.ac.th

**Keywords:** incubation temperature, relative humidity, egg hatchability pattern, two-spotted cricket (*Gryllus bimaculatus*), house cricket (*Acheta domesticus*)

## Abstract

**Simple Summary:**

Simple Summary: Cricket farming requires high proportions of nymph hatching and a shorter period from incubation to hatching to increase productivity and lower production costs, resulting in optimized conditions and productivity. Two-spotted (*Gryllus bimaculatus*) and house (*Acheta domesticus*) crickets are two important cricket types in domesticated and industrial production. When the eggs of the two cricket types were subjected to a low to high incubation temperature (23–33 °C) with a constant relative humidity (RH) of 70%, the two-spotted crickets exhibited faster hatching (emergence) than the house crickets. Additionally, the onset of hatching decreased with higher incubation temperatures for both types of crickets. Afterward, RH levels varied during egg incubation. It was found that an optimum incubation temperature of 31 °C and 70% RH produced a hatching rate of about 80% and 6 days for the start of hatching for two-spotted crickets, while an optimum incubation temperature of 30 °C and 65–75% RH provided a daily peak hatching rate of about 66% and 12 days for the start of hatching for house crickets. Further studies are required to better clarify the conditions that may influence hatchability and productivity.

**Abstract:**

This study aimed to determine the influence and optimal conditions of incubation temperature and relative humidity (RH) on the egg hatchability patterns of two-spotted (*Gryllus bimaculatus*) and house (*Acheta domesticus*) crickets. Experiment I involved 100 cricket eggs per hatching box for each species, with six replications for each controlled incubation temperature of 23, 25, 27, 29, 30, 31, 32, and 33 °C at 70% RH. Experiment II used all the same procedures as Experiment I, except for incubation temperatures of 29, 30, 31, and 32 °C tested with varied RH levels of 65%, 70%, and 75%. In Experiment I, two-spotted crickets (9.47 ± 1.99 days) exhibited faster hatching than house crickets (13.70 ± 2.78 days). Additionally, the onset of hatching decreased with higher incubation temperatures for both types of crickets. In Experiment II, an incubation temperature of 31 °C and 70% RH resulted in a hatching rate of 79.75% for two-spotted crickets, with hatching beginning in 6 days. For house cricket eggs, the optimal conditions of 30 °C and 65–75% RH led to a peak daily hatching rate of 62.00–65.50% and hatching onset in 12 days. Thus, this study established the optimal incubation temperature and RH for egg hatching of two-spotted and house crickets.

## 1. Introduction

Edible insects represent a novel alternative food source that supports sustainability (i.e., [1,2,3,4,5]). Insect farming plays a crucial role in achieving sustainability goals [6]. In cricket farming, *G. bimaculatus* is noted for its economic impact, while *A. domesticus* is preferred for its more acceptable taste [7]. Cricket farming is currently recognized as an alternative approach to animal production, named mini-livestock farming. It holds significant potential for appreciably reducing the global environmental impact compared to traditional livestock used for protein sources, such as chicken, goat, pork, and beef (i.e., [8,9,10]). In addition, cricket farming can alleviate poverty among farmers and stimulate the development of various related industries, particularly within cricket production chains.

Currently, cricket farming is on the rise. Like livestock production, there are three main factors—variations in animals, production resources, and farm management—that affect production efficiency. A high proportion of low body weight in adults and immature crickets in each generation of production results in lower efficiency of cricket production and lower selling prices. The variation in body size of crickets can be caused by several factors, including high variability in the onset of hatching, inadequate feed and feeding management, and suboptimal rearing environmental conditions. From the literature review of earlier reports [6,11,12,13,14,15,16], it was found that higher incubation temperatures for cricket eggs resulted in a shorter onset of hatching. From an animal science point of view, a higher proportion of larvae hatching from eggs, a shorter onset of hatching, and a shorter duration to complete hatching per generation of production will result in a multiplier effect: higher production generations per year and a better chance of achieving uniformity among crickets. However, there is still limited understanding of the factors influencing cricket egg hatching, which directly affects production quantities. According to earlier reports [6,11,12,13,14,16], different incubation temperature levels have been shown to influence the onset of hatching (development time), with variations observed among cricket species. In the meantime, there have been few reports on hatchability (overall hatching rate) and the number of eggs that hatch per batch [6]. These reports indicate hatchability rates of 60.23% for two-spotted crickets [17], 63.30–95.00% for *Modicogryllus conspersus* [6], and 70.00–86.25% for *Gryllus mitratus* [14]. For insects, many eggs must absorb water before they can complete their development. Sufficient moisture in the environment can prevent death through desiccation [18]. Eggs are the most sensitive to desiccation during the first several days after being laid and during the period of water absorption [19]. Based on available information, the relative humidity required for hatching eggs ranges between 65 and 80%, with an ambient temperature of 26 °C [20]. High relative humidity can promote the growth of mold and bacteria, resulting in lower hatchability [15]. More information about the patterns of hatchability and the crucial factors of incubation temperature and relative humidity is essential for efficient production. Understanding these factors will help optimize conditions for better hatching rates and overall productivity in cricket farming.

Currently, there is limited information concerning the hatchability of eggs for *G. bimaculatus* and *A. domesticus*, possibly due to exposure to different ambient incubation temperatures; no studies exist on the relative humidity factor. Therefore, the current study aimed to determine the influence and optimal conditions of incubation temperature and relative humidity on the egg hatchability patterns of two-spotted (*G. bimaculatus*) and house (*A. domesticus*) crickets.

## 2. Materials and Methods

### 2.1. Incubation Unit for Cricket Egg Hatching

**Figure 1:**
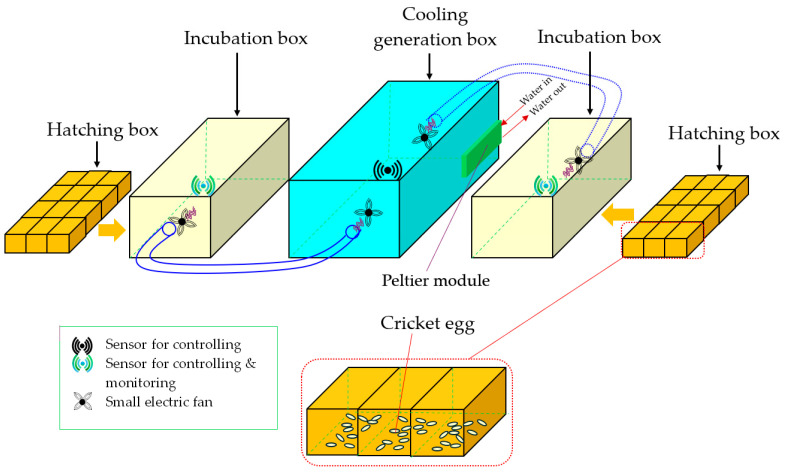
The cricket egg-hatching incubation unit comprises one cooling generation box equipped with a Peltier module and two incubation boxes. The hatching boxes containing cricket eggs are placed into the incubation boxes during the incubation process.

The cricket egg-hatching incubation unit comprises two main types of boxes: one cooling generation box and two incubation boxes (Figure 1). The structure of the cooling generation box is rectangular with dimensions of 24 (width) × 43 (length) × 20 (height) cm. The box has areas for installing one set of Peltier plates and heat sinks (thermoelectric cooling module). A motor pump is used to pump water through rubber tubes into the aluminum block, which is in contact with the low-temperature side of the Peltier plates, and the high-temperature side is in contact with the heat sink, dissipating heat with a fan. There is also an air outlet for ventilation with another fan. Temperature is monitored and controlled by temperature sensors. Water temperature is reduced by one set of the Peltier plates. Aluminum blocks with fans installed for heat dissipation are in contact with the low-temperature side, allowing water to flow and cool down. This works in conjunction with the motor pump. On the high-temperature side, heat sink plates dissipate heat with fans, maintaining the desired temperature. For the incubation box, a temperature control system is utilized with an airflow fan blowing cool air from the cooling generation box. Sensors in the cooling generation box are used to monitor potential issues, while sensors in the incubation box measure temperature levels and control the operation of the airflow fan from the cooling generation box into the incubation box. Specifically, if the temperature in the incubation box exceeds the desired level, the airflow fan will draw cool air from the cooling generation box into the incubation box until the temperature in the incubation box falls below the desired level, at which point the fan will stop operating. Furthermore, this temperature control system can monitor and control operations remotely (via an online application) as well as record the relative humidity levels inside the box with humidity sensors.

### 2.2. Cricket Egg Production

To produce cricket eggs for the current study, cricket eggs of the two-spotted (*G. bimaculatus*) and house (*A. domesticus*) species were purchased separately from a commercial cricket breeder in Thailand (Somjainuk cricket’s farm, Nakhon Sawan, Thailand; https://www.facebook.com/somjainukcf/). All crickets were reared at a farm facility belonging to the Animal Science Division, Department of Animal Production Technology and Fisheries, School of Agricultural Technology at King Mongkut’s Institute of Technology Ladkrabang, Thailand. Cricket rearing and management used the procedure mentioned in an earlier report [21], except for half the size of rearing boxes, including house crickets, and sample collection. Ambient temperature and relative humidity for cricket rearing to produce eggs for Experiment I ranged from 28.6 °C to 34.3 °C (average of 31.45 °C) and from 51.0% to 86.05% (average of 72.35%), respectively. Briefly, a commercial cricket feed (Pure Pride^®^; PP feed, the TFMS (Saraburi) Co., Ltd., Samutsakorn, Thailand) contained 21.15% crude protein. Egg board cartons were continuously arranged and placed in the rearing boxes. Feed and drinking water were offered ad libitum. When approximately 95% of the crickets had reached full growth with the appearance of wings, a tray with a moisturized mixture of coconut coir was provided, allowing some female crickets of each cricket species to lay eggs for 24 h. The cricket eggs obtained from all rearing boxes of each species were then mixed and prepared for further hatching studies. The leftover cricket eggs were incubated under natural conditions. After hatching, the crickets were reared using the same procedure as described for the first round of cricket production in order to collect cricket eggs for the next phase of the hatching trial.

### 2.3. Cricket Egg Incubation

**Figure 2:**
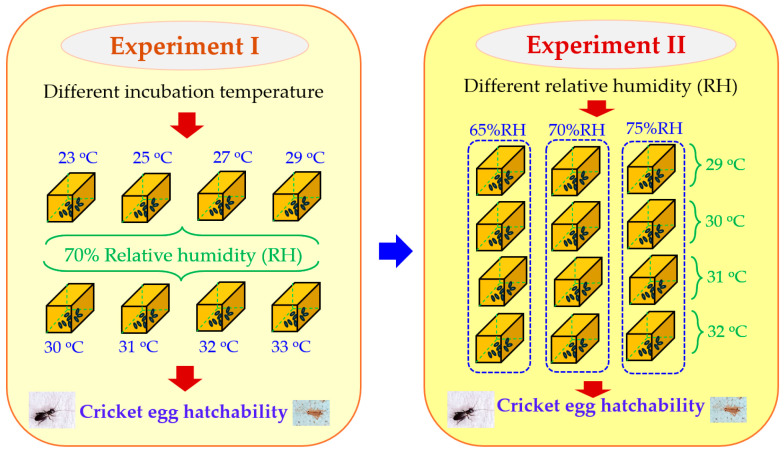
Scheme of Experiments I and II for the current study. Experiment I: Different ambient incubation temperature levels were applied to hatch both studied cricket species. Experiment II: The results indicating the possible optimum incubation temperature levels from Experiment I were selected to test the influence of ambient relative humidity in both studied cricket species.

For Experiment I (Figure 2), the eggs of two-spotted and house crickets laid on the same day were separated from the coconut coir using cotton buds to remove them. They were then placed in a fine mesh sieve and washed with tap water, followed by rinsing with normal saline solution. The eggs were placed in transparent boxes for incubation, called hatching boxes. Before transferring them to the hatching boxes, naturally brown recycled paper, processed through a heat treatment process and without bleaching agents, was laid on the floor of the hatching boxes. The base lining of the hatching boxes (Figure 1) was smooth, with a snug-fitting lid. The dimensions of the hatching boxes were 7 cm (width) × 9 cm (length) × 3.5 cm (height). Every edge of the top of each hatching box was lined with smooth tape, approximately 0.5 cm from the top edge, to prevent crickets from being able to crawl out of the box. All hatching boxes used in this study were sterilized. A total of 100 cricket eggs per hatching box for each cricket species were placed in the boxes, with 6 replications for each controlled incubation temperature level. Then, 4 mL of normal saline solution was added into each box, and the lid was closed tightly to maintain the relative humidity level at 70%. A natural photoperiod of 12 h of light to 12 h of darkness was provided. A sensor for temperature and relative humidity was equipped in each hatching box to monitor the actual environmental temperature and relative humidity. The hatching boxes containing cricket eggs were then placed into the incubation box set to incubation temperature levels of 23, 25, 27, 29, 30, 31, 32, and 33 °C. The 6 replications of both two-spotted and house crickets were placed in the same incubation box.

For Experiment II (Figure 2), similar procedures and management as in Experiment I were used, except that the cricket eggs were obtained from the second round of cricket production. Ambient temperature and relative humidity for cricket rearing to produce eggs for Experiment II ranged from 28.6 °C to 35.5 °C (average of 31.86 °C) and from 55.00% to 83.00% (average of 69.99%), respectively. Additionally, the incubation temperature levels, selected based on the results of Experiment I and a literature review (Table 1), were set at 29, 30, 31, and 32 °C. Further details explaining the selection of these incubation temperatures are provided in the discussion. The relative humidity levels varied at 65%, 70%, and 75%, with four replications for each combination of incubation temperature and relative humidity. Normal saline solutions at volumes of 2, 4, and 6 mL were added to the hatching boxes to achieve relative humidity levels of 65%, 70%, and 75%, respectively.

### 2.4. Data Recording

The results of this study were recorded by counting the number of cricket nymphs that hatched from the eggs twice daily at 9:00 a.m. and 5:00 p.m. The counts from both times were combined to calculate the daily hatching rate (%) of two-spotted and house crickets. The dates when hatching occurred were recorded. The hatched cricket nymphs, counted each day, were removed from the hatching box, placed on ice for 15 min, and then preserved in a 70% alcohol solution to maintain their condition and verify the hatching count. The hatching rate was calculated using the following formulas:

Daily hatching rate (%) = (Number of crickets hatched daily in each hatching box/Number of eggs at the start) × 100(1)

Overall hatching rate (%) = (Number of all crickets hatched in each hatching box/Number of eggs at the start) × 100.(2)

Peak daily hatching rate (%) was defined as the maximum daily hatching rate. The onset of hatching and the day of peak daily hatching were defined as the day the first hatching occurred and the day of maximum daily hatching rate observed after incubation, respectively. The duration from onset to the last day of hatching (days) was also determined.

### 2.5. Statistical Analysis

Data were analyzed using cricket species and incubation temperature as the main effects for Experiment I and using incubation temperature and relative humidity as the main effects for Experiment II via the General Linear Model (GLM). A two-factor analysis of variance (ANOVA) was applied. One-way ANOVA was used to detect differences among the incubation temperatures for each cricket species in Experiment I. Differences between the groups were determined using the Tukey test. The relationship between incubation temperature and hatchability parameters was described by regression equations. The correlation coefficient between the onset of hatching and the day of peak daily hatching was determined using bivariate correlation. All statistical analyses were performed using the SPPP version 28.0 (IBM SPSS Statistic^®^, SPSS Inc., Chicago, IL, USA).

## 3. Results

### 3.1. Impact of Incubation Temperature on Hatchability Pattern (Experiment I)

**Figure 3:**
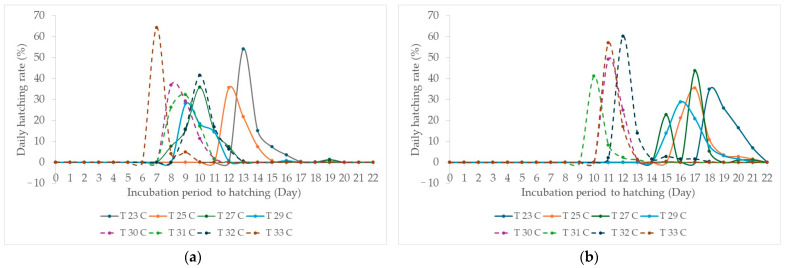
Mean daily hatching rates (%) of two-spotted (**a**) and house (**b**) crickets each day after being exposed to different incubation temperatures.

For Experiment I, the overall measured ambient temperatures were 23.05 ± 0.15, 25.03 ± 0.13, 27.07 ± 0.20, 29.07 ± 0.19, 30.02 ± 0.18, 30.98 ± 0.14, 32.08 ± 0.15, and 33.05 ± 0.11 for incubation temperature settings of 23, 25, 27, 29, 30, 31, 32, and 33 °C, respectively. The average daily hatching rate for each incubation temperature, with a constant relative humidity of 70% (70.48 ± 0.46% as measured), was calculated and illustrated in Figure 3, separated by studied cricket species. There was an overlap in the daily hatching rate curves at incubation temperatures of 27, 29, and 32 °C (Figure 3a). There was a trend of slower hatching for two-spotted cricket eggs incubated at 23 and 25 °C, as well as a trend of faster hatching for eggs incubated at 30, 31, and 33 °C (Figure 3a). In the meantime, this study indicated that incubation temperatures of 30–33 °C resulted in faster hatching for house crickets (Figure 3b) compared to temperatures of 23–29 °C.

The cricket egg hatching pattern was described by overall hatching rate, peak daily hatching rate, the onset of hatching, day of peak daily hatching, and the duration from onset to the last hatching, as shown in Table 2. A two-factor analysis of variance was applied to these hatchability parameters using cricket species and incubation temperature as the main effects (Table 2). The analysis showed a significant influence (*p* < 0.01) of incubation temperature on all hatchability parameters. The influence of cricket species was significant for the onset of hatching (*p* < 0.001) and the day of peak daily hatching (*p* < 0.001). Additionally, the interaction between cricket species and incubation temperature significantly affected the day of peak daily hatching rate (*p* < 0.05), onset of hatching (*p* < 0.001), and day of peak daily hatching (*p* < 0.001). Due to the presence of interaction, the different effects among incubation temperatures within each cricket species were detected.

For two-spotted crickets, no significant differences (*p* > 0.05) were found in the overall hatching rate and the duration from onset to the last hatching (Table 2), with averages of 76.98% and 4.64 days, respectively. The lowest (*p* < 0.05) and highest (*p* < 0.05) of peak daily hatching rates were 40.17% and 64.33% at incubation temperatures of 25 and 33 °C, respectively. The onset of hatching (ranging from 13.00 to 7.00 days) and day of peak daily hatching (ranging from 13.00 to 7.00 days) gradually decreased (*p* < 0.05) with higher incubation temperatures (from 23 to 33 °C), except for a fluctuation at 32 °C.

For house crickets, the lowest (*p* < 0.05) overall hatching rate was 70.50% at an incubation temperature of 31 °C, while the highest (*p* < 0.05) overall hatching rate was 84.50% at 32 °C. The second highest (*p* < 0.05) rate was 84.00% at 23 °C. Peak daily hatching rates at 25 °C and 29 °C, both 35.5%, were lower (*p* < 0.05) than those at 32 °C and 33 °C, which were 64.83% and 59.67%, respectively. Regarding the onset of hatching (ranging from 18.00 to 10.00 days) and the day of peak daily hatching (ranging from 18.33 to 10.75 days), there was a gradual decrease (*p* < 0.05) with higher incubation temperatures from 23 to 31 °C, followed by a slight increase at 32 °C and 33 °C. The shortest (*p* < 0.05) duration from onset to the last hatching was observed at 30 °C (2.67 days), with the second shortest (*p* < 0.05) duration at 33 °C (2.83 days). The longest (*p* < 0.05) duration was observed at 29 °C (6.00 days).

The linear regression relationship between the incubation temperature and hatchability pattern of two-spotted and house crickets was analyzed using linear regression to determine causality (Table 3). For two-spotted crickets, no significant relationships (*p* > 0.05) were found between incubation temperature and overall hatching rate, peak daily hatching rate, or duration from the onset to the last hatching. However, the onset of hatching (*R*^2^ = 0.784) and the day of peak daily hatching (*R*^2^ = 0.745) were influenced by incubation temperature (*p* < 0.001), with satisfactory *R*^2^ values above 0.65 [24]. For house crickets, no significant relationships (*p* > 0.05) were found between incubation temperature and overall hatching rate, or the duration from onset to the last hatching. However, peak daily hatching rate (*R*^2^ = 0.260), the onset of hatching (*R*^2^ = 0.813), and the day of peak daily hatching (*R*^2^ = 0.791) were influenced by incubation temperature (*p* < 0.001). For house crickets, no significant relationships (*p* > 0.05) were found between incubation temperature and overall hatching rate or the duration from onset to the last hatching. However, the peak daily hatching rate (*R*^2^ = 0.260), the onset of hatching (*R*^2^ = 0.813), and the day of peak daily hatching (*R*^2^ = 0.791) were influenced by incubation temperature (*p* < 0.001), with satisfactory *R*^2^ values above 0.65 with satisfied *R*^2^ value of above 0.65 [24]. The incubation temperature could be classified into two classes: <30 °C and ≥30 °C. Incubation temperatures of 29, 30, 31, and 32 °C were selected for further study concerning the impact of relative humidity in Experiment II.

In Table 1, a literature review on the onset of hatching under different incubation temperatures and relative humidity is illustrated. The key information from the review indicates that an incubation temperature that is too low results in a prolonged onset of hatching, while too high an incubation temperature results in a shorter onset of hatching, with a non-linear relationship showing very minor changes in the onset. The study by Odhiambo et al. [11] showed a trend of lower incubation temperatures required and a longer onset of hatching for house crickets compared to two-spotted crickets under the same environmental conditions, except for uncontrolled relative humidity.

### 3.2. Impact of Incubation Temperature and Relative Humidity on Hatchability Pattern (Experiment II)

For Experiment II, the overall measured ambient temperatures were 29.01 ± 0.19, 29.99 ± 0.14, 31.02 ± 0.15, and 32.05 ± 0.12 °C for incubation temperature settings of 29, 30, 31, and 32 °C, respectively. The overall ambient relative humidities were 65.55 ± 0.20%, 70.66 ± 0.34%, and 75.58 ± 0.63% for relative humidity settings of 65%, 70%, and 75%, respectively. From Table 4, there was no significant impact of incubation temperature on the overall hatching rate (*p* > 0.05) for two-spotted cricket egg incubation, but relative humidity did have a significant effect (*p* < 0.05). Conversely, incubation temperature for the cricket eggs significantly influenced the peak daily hatching rate (*p* < 0.05), while relative humidity had no significant impact on it (*p* > 0.05). Both incubation temperature and relative humidity had significant effects on the onset of hatching, the day of peak daily hatching, and the duration from onset to the last hatching (*p* < 0.001), except for the effect of relative humidity on the duration from onset to the last hatching, which was significant at *p* < 0.05. In addition, an interaction between incubation temperature and relative humidity (*p* < 0.05) was found for the onset of hatching and the day of peak daily hatching. The highest (*p* < 0.05) overall hatching rate (79.75%) was found at an incubation temperature of 31 °C with 70% relative humidity. The highest (*p* < 0.05) peak daily hatching rate (62.50%) was found at an incubation temperature of 32 °C with 65% relative humidity. The onset of hatching was the shortest (*p* < 0.05), at 6 days, for the incubation temperature of 31 °C. The shortest (7.75 days, *p* < 0.05) and second shortest (8.00 days, *p* < 0.05) durations to peak daily hatching were found for an incubation temperature of 31 °C with 75% and 70% relative humidity, respectively. The shortest (2.50 days, *p* < 0.05) and second shortest (2.75 days, *p* < 0.05) durations from onset to the last hatching were found for an incubation temperature of 32 °C with 65% relative humidity and 31 °C with 75% relative humidity, respectively.

For house crickets (Table 4), there was no significant impact of either incubation temperature or relative humidity on the overall hatching rate (*p* > 0.05), though interaction between these two factors was found (*p* < 0.05). The incubation temperature for cricket eggs had a significant impact (*p* < 0.001) on peak daily hatching rate, the onset of hatching, the day of peak daily hatching, and the duration from onset to the last hatching. However, there was no significant impact (*p* > 0.05) of relative humidity or interaction between these two factors, except for their interaction affecting the duration from onset to the last hatching (*p* < 0.05).

The highest (*p* < 0.05) peak daily hatching rates ranged from 62.00% to 65.50% and 64.75% to 75.50% when cricket eggs were exposed to incubation temperatures of 30 °C and 31 °C, respectively, across the three studied relative humidity levels.

The shortest duration (12.00 days, *p* < 0.05) and the range of the shortest durations (12.00–12.25 days, *p* < 0.05) were observed in crickets exposed to an incubation temperature of 30 °C across the three studied relative humidity levels. The ranges of the shortest duration (*p* < 0.05) from onset to the last hatching were 1.00–1.75 days and 1.75–2.25 days when the cricket eggs were exposed to incubation temperatures of 30 and 31 °C, respectively, across the three studied relative humidity levels.

**Figure 4:**
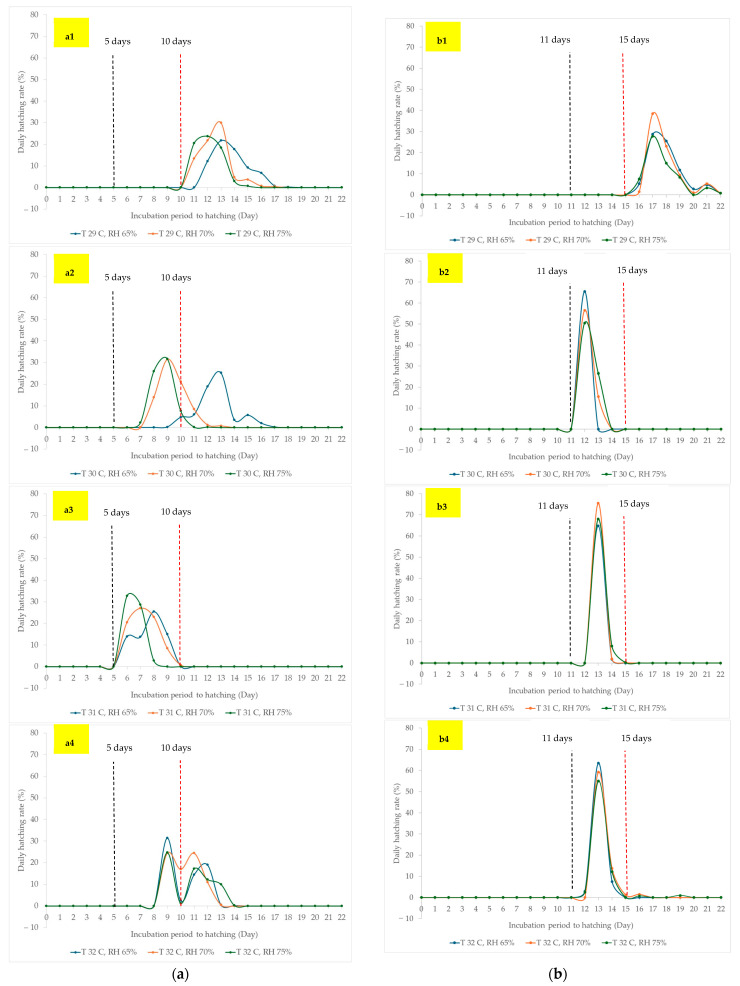
Mean daily hatching rates (%) of two-spotted (**a**) and house (**b**) crickets each day after being exposed to different levels of relative humidity and incubation temperatures of 29 °C (**a1**,**b1**), 30 °C (**a2**,**b2**), 31 °C (**a3**,**b3**), or 32 °C (**a4**,**b4**).

Given the presence of an interaction between incubation temperature and relative humidity in several hatching parameters, the daily hatching rate was plotted across the days of incubation, classified by each incubation temperature (Figure 4). For the two-spotted crickets (Figure 4a), a relative humidity of 65% at incubation temperatures of 29 and 30 °C delayed the onset of hatching by a few days and prolonged the duration of hatching, especially at an incubation temperature of 30 °C. When the incubation temperatures were 31 and 32 °C, the onset of hatching was the same for each of the three different humidity levels. However, relative humidity produced different patterns in the daily hatching curve. Exposing eggs to an incubation temperature of 31 °C with three levels of relative humidity resulted in all eggs starting to hatch on day 6 and completing hatching by day 10 of incubation. It was clear for the house crickets that an incubation temperature of 30–32 °C resulted in almost all eggs hatching at 12–15 days after incubation, with faster hatching compared to an incubation temperature of 29 °C (Figure 4b). There was a small peak in the daily hatching rate just before the end of hatching for both incubation temperatures of 29 and 32 °C.

## 4. Discussion

The current study used eggs from both two-spotted and house crickets laid on the same day for both Experiment I and Experiment II. The cricket eggs were obtained from crickets that were reared using a commercial diet and standard farm management practices. Therefore, several of the factors that could potentially affect embryo development were either controlled or mitigated. Additionally, all studied cricket eggs were randomly selected from a pooled and well-mixed batch. Contamination from bacteria or mold was reduced through washing with water and rinsing with a normal saline solution.

Overall hatching rates, peak daily hatching rates, and duration from onset to the last hatching found in the current study fluctuated and showed inconsistent changes with the incubation temperature (Table 2).

These variations would be mainly contributed by the high standard deviation in each incubation temperature for each measured parameter. As there are rather scarce reports about hatchability (referred to as overall hatching rate in the current study), the hatchability of 60.23% for two-spotted crickets (at presumably 74.9–86.20% relative humidity) [20], 63.30–95.00% (at high relative humidity by daily spraying) for *Modicogryllus conspersus* [6], and 70.00–86.25% (at 68.82–69.04% relative humidity) for *Gryllus mitratus* [14] were reported. Based on these few reports, relative humidity might play a role in the hatchability of cricket eggs.

At the overall incubation temperature in this study, there was an average overall hatching rate of 76.98 ± 10.48% for two-spotted crickets and 77.33 ± 7.75% for house crickets. These results indicated comparable hatchability to those reported earlier. However, the study by Ssepuuya et al. [6] showed that the peak overall hatching rate (95%) occurred at an incubation temperature of 28 °C, decreasing by 23.3% and increasing by 10.6% for every 2 °C below and above 28 °C, respectively. This pattern was not found in the current study but showed inconsistency for change in the overall hatching rate with incubation temperature. This discrepancy might be due to three possible factors, comprising (1) different properties of chorion [18] and breakdown of food reserves [25], (2) water loss and absorption of water [18], and (3) unhatched eggs eaten by newly hatched nymphs [26]. When the average daily hatching rate for each incubation temperature was illustrated as a line curve (Figure 3), the hatchability pattern was quite similar but differed in the value of amplitude and width of the line curve. Thus, depicting the daily hatching rate pattern could be an alternative way to describe the hatching ability of cricket eggs. According to a study by Tobing et al. [15], cricket egg quality decreases under unsuitable environmental conditions. Low humidity can cause damage to the eggs, while high humidity can lead to fungal overgrowth or disease on the eggs [15].

There was no significant relationship between incubation temperature and the overall hatching rate, peak daily hatching rate, or the duration from onset to the last hatching. A possible explanation could be similar to those mentioned earlier for the overall hatching results, as the peak daily hatching rate and duration from onset to the last hatching are likely consequences of the hatching process. Although a relationship was found for the peak daily hatching rate of the house crickets, the R^2^ value (0.260) was rather low, probably due to the high variation in peak daily hatching rates. The linear regression relationship between incubation temperature and hatchability patterns of two-spotted and house crickets showed that an increase in incubation temperature (from 23 °C to 33 °C) would decrease the onset and peak daily hatching days for both two-spotted and house crickets. The correlation coefficient between the onset of hatching and the day of peak daily hatching was very strong [27] for both two-spotted crickets (*r* = 0.965, *p* < 0.001) and house crickets (*r* = 0.966, *p* < 0.001). The average values for both the onset of hatching and the day of peak daily hatching were close to each other (within 1.7 days) or identical, indicating a hatching pattern similar to a bell curve (Figure 3). The line curve of the daily hatching rate in this study was comparable to the frequency histogram of hatching [28,29], making it suitable for gaining insight into cricket egg hatching.

For this study, the linear regression equations for the relationship between incubation temperature (23–33 °C) with a relative humidity of 60–90% and the onset of hatching were Y = 24.620 − 0.527X for two-spotted crickets and Y = 35.187 − 0.750X for house crickets, where Y is the onset of hatching (day) and X is the incubation temperature (°C). This indicates that increasing the incubation temperature by 1 °C shortens the time required for the larvae to hatch from eggs by approximately 0.527 days for two-spotted crickets and 0.750 days for house crickets.

Based on the results of Odhiambo et al. [11], using an incubation temperature range of 18–38 °C with a relative humidity of 70%, the means of hatching onset (development time in days) were used to derive equations for the linear regression relationship. The equations were Y = 71.202 − 1.818X and Y = 66.055 − 1.629X for two-spotted and house crickets, respectively. This discrepancy implies that cricket species and other factors impact the onset of hatching. There were four other reports available on the relationship between incubation temperature and the onset of hatching. Hermansa et al. [13] reported the equation Y = 74.655 − 2.0574X (incubation temperature of 20–29 °C, relative humidity of 65 ± 5%) for *Gryllus assimilis*. Srygley [16] studied *Anabrus simplex* by exposing cricket eggs to incubation temperatures ranging from 10 to 26 °C (moist sand with 25% fully saturated). Recalculation of the equation from the supplementary data resulted in Y = 30.710 − 1.076X. Modifications from the reports of Magara et al. [12] for *G. bimaculatus* and Ssepuuya et al. [6] for *M. conspersus* provided the equations Y = 43.147 − 1.181X (incubation temperature of 25–32 °C, relative humidity of 70 ± 5%) and Y = 37.623 − 0.907X (incubation temperature of 26–34 °C, high relative humidity by water spraying), respectively. These three reports indicate a similar impact of increasing incubation temperature on shortening the onset of hatching but at different rates, which might be due to different cricket species, incubation temperature ranges (including very low or high temperatures), and relative humidity conditions. For *G. bimaculatus*, the time required for larvae to hatch from eggs in this study (0.527 days per 1 °C) and the study by Magara et al. [12] (1.181 days per 1 °C) differs by about twofold. These differences might be caused by variations in cricket species, cricket egg quality, environmental gas ventilation, or relative humidity in the experiments. In principle, the rate of water loss from newly laid insect eggs is often very low, especially in eggs of species that do not take up water during development [18]. In the cricket (*Allonemobius fasciatus*), water is taken up at an earlier stage of development at 30 °C than at 20 °C [18]. In the cricket (*Teleogryllus*), the chorion remains permeable at all times, but initial water uptake is prevented. This results in an increase in hydrostatic pressure, which may then be responsible for preventing further water uptake [18]. An earlier report by Rouault et al. [25] found that reducing the development rate of the embryo could be caused by a slowdown in enzymatic activity and a lower rate of breakdown of food nutrient reserves. The duration of development decreases as temperature increases [18].

In the current study, the floor of the hatching boxes was always dry at an incubation temperature of 33 °C in Experiment I, and condensed water was found on the lid. In contrast, only a low occurrence of condensed water was found at 32 °C, implying a more desiccated environment. According to the results of Magara et al. [12], there was a drop in egg development at an incubation temperature of approximately 33–34 °C. No difference in overall hatching rate (hatchability) or onset of hatching (development duration) was found for the incubation temperatures of 32 °C and 34 °C in the study by Ssepuuya et al. [6]. These findings led to the exclusion of the incubation temperature of 33 °C for Experiment II. Additionally, incubation temperatures below 29 °C in the present study were excluded from Experiment II due to their lower potential to reduce the onset of egg hatching, especially for house crickets. From the results of Odhiambo et al. [11], the development of *G. bimaculatus* and *A. domesticus* from egg to adult occurs between the thermal ranges of 22–30 °C and 26–34 °C, respectively. An incubation temperature of 29 °C was found and presumed to be a critical level for shortening the onset of cricket egg hatching for both kinds of crickets in the current study, which falls within the recommended ranges of ambient temperature for both cricket species reported by Odhiambo et al. [11]. At the incubation temperature of 29–32 °C selected for Experiment II, environmental relative humidity might be a factor in preventing desiccation during cricket egg hatching. For Experiment II, the effects of relative humidity varied at 65%, 70%, and 75% under different incubation temperature levels of 29, 30, 31, and 32 °C, as shown in Table 4 and Figure 4. Incubation temperature levels had an impact on the hatchability pattern, except for the overall hatching rate, for both two-spotted and house crickets. Meanwhile, different relative humidity levels influenced the hatching pattern, except for the peak daily hatching rate of two-spotted crickets, whereas no impact on the hatching pattern was found in house crickets.

The interaction between incubation temperature and relative humidity was found to affect the onset of hatching and the day of peak daily hatching for two-spotted crickets. This indicates that relative humidity and incubation temperature influence the onset and peak days of hatching in different ways. From Figure 4(a3), it is clear that the combination of an incubation temperature of 31 °C and all three levels of relative humidity resulted in a shorter onset and peak day of hatching compared to 29 °C. Under the ambient conditions in this study, the eggs of two-spotted crickets exposed to an incubation temperature of 31 °C and 70% relative humidity achieved an overall hatching rate of 79.75%, with an onset of hatching in 6 days and a duration of approximately 2–4 days from the first to the last hatching. However, exposure to 65% and 75% relative humidity could also shorten the onset of hatching, though with a lower overall hatching rate. For house crickets, eggs exposed to an incubation temperature of 30 °C and all three levels of relative humidity showed a peak daily hatching rate of 62.00–65.50%, with an onset of hatching in 12 days, a daily peak hatching period of 12.00–12.25 days, and a duration of approximately 1–2 days from the first to the last hatching. Although there was no difference in the overall hatching rate, the cumulative daily hatching rate could explain the findings. The interaction between incubation temperature and relative humidity for overall hatching rate and duration from onset to the last hatching might be due to the small peak of daily hatching rates at 29 °C and 32 °C. This is the first report that studied the influence of relative humidity on cricket egg incubation, which partly illustrated suitable ambient temperature and relative humidity, as suggested by Ssepuuya et al. [6]. However, a wider range and larger scale of relative humidity, i.e., 60%, 70%, 80%, and 90% should be studied further to identify more optimal conditions for incubation temperature and relative humidity.

From the point of view of animal production, a high proportion of nymphs hatch, and the short onset of hatching leads to higher numbers of animals and higher generations per year, resulting in more animal yields for cricket farming. However, high growth performance and higher survival rates from stronger animals are crucial to the success of producing animals efficiently and sustainability. According to Ssepuuya et al. [6], high ambient temperatures may decrease food reserves in eggs, resulting in less energy available for growth and lower body weight in newly hatched nymphs. Low-weight nymphs at hatching are a result of their incomplete maturity, which consequently leads to weakness and higher mortality. The study by Odhiambo et al. [11] found that, in both two-spotted and house crickets, adult longevity had a positive relationship with the onset of hatching (development time), while a negative relationship was found for fecundity and body weight. However, Magara et al. [12] reported that female two-spotted crickets had the highest and second-highest body weight when reared at constant ambient temperatures of 32 °C and 27 and 30 °C, respectively. Male two-spotted crickets had the highest weight when reared at constant ambient temperatures of 27, 30, 32, and 35 °C. This discrepancy could be explained by the feed availability and intake of nymph crickets during the first three weeks after hatching. Earlier reports indicated that there was no weight increase during the first three weeks (possibly due to acclimation to rearing conditions and the offered feed) for *M. conspersus* [6]. Additionally, the growth pattern of two-spotted crickets changed on day 18, suggesting that a two-phase feeding regimen should be used to rear the crickets [30]. Therefore, feed and feeding management for crickets should be studied further and properly applied to ensure suitable production.

## 5. Conclusions

Incubation temperature with 70% ambient relative humidity conditions for the eggs of two-spotted and house crickets resulted in different hatchability patterns for each cricket species. At higher incubation temperatures, two-spotted crickets exhibited a lower onset of hatching (emergence) and required a longer time compared to house crickets. When high incubation temperature levels with varied ambient relative humidity levels were investigated, the two cricket species exhibited different responses. For two-spotted crickets, the optimal conditions were an incubation temperature of 31 °C and 70% relative humidity, resulting in an overall hatching rate of 79.75%. The onset of hatching occurred in 6 days, with a duration of approximately 2–4 days from the first to the last hatching, presenting an interaction effect between incubation temperature and relative humidity. For house crickets, the optimal conditions were an incubation temperature of 30 °C and all three levels of relative humidity tested (65%, 70%, and 75%), resulting in a peak daily hatching rate of 62.00–65.50%. The onset of hatching occurred in 12 days, with a peak daily hatching period of 12.00–12.25 days and a duration of approximately 1–2 days, without the impact of varied relative humidity levels. However, further studies are required to refine these conditions and explore additional factors that may influence hatchability and productivity.

## Figures and Tables

**Table 1:** Results of a literature review for onset of hatching under different incubation temperatures and relative humidity.

Reference Source	Cricket Species	Incubation Temperature (°C)	Relative Humidity (%)	Onset of Hatching (day)	Remark
Odhiambo et al. [11]	*Acheta domesticus*	22–30	60–90	10.21–35.41	Excluded 18 °C, 34 °C, and 38 °C
Douan et al. [22]	*Acheta domesticus*	28 ± 2	50 ± 7	16.00	
Odhiambo et al. [11]	*Gryllus bimaculatus*	26–34	60–90	9.45–10.71	Excluded 18 °C, 22 °C, and 38 °C
Magara et al. [12]	*Gryllus bimaculatus*	20–32	70 ± 5	5.93–34.13	Excluded 35 °C and 37 °C
Donoughe and Extavour [23]	*Gryllus bimaculatus*	29	High	12.00	Damp sand as media
Hermansa et al. [13]	*Gryllus assimilis*	20–29	65 ± 5	15.10–33.60	
Brata et al. [14]	*Gryllus mitratus*	26.18–26.39	68.82–69.04	5.00–10.00	
Tobing et al. [15]	*Gryllus mitratus*	N.A.	High	13.00–14.00	Media for hatching study
Srygley [16]	*Anabrus simplex*	10–26	High	8.80–22.85 (recalculated)	Moist sand with 25% fully saturated
Ssepuuya et al. [6]	*Modicogryllus conspersus*	26–34	High	7.07–14.40 (calculated)	Water spraying

N.A. = no available information.

**Table 2:** Egg hatchability pattern (mean ± SD) of the experimental two-spotted and house crickets at different incubation temperature levels and a controlled relative humidity of 70% (Experiment I).

Incubation Temperature	Overall Hatching Rate (%)	Peak Daily Hatching Rate (%)	Onset of Hatching (Day)	Day of Peak Daily Hatching (Day)	Duration from Onset to the Last Hatching (Day)
*Two-spotted cricket*					
23	81.83 ± 9.79 ^a^	54.17 ± 11.96 ^ab^	13.00 ± 0.00 ^e^	13.00 ± 0.00 ^d^	6.00 ± 1.55 ^a^
25	66.00 ± 13.96 ^a^	40.17 ± 6.49 ^a^	12.00 ± 0.00 ^d^	12.17 ± 0.41 ^d^	4.67 ± 1.97 ^a^
27	82.33 ± 2.73 ^a^	47.17 ± 11.51 ^ab^	9.17 ± 0.98 ^c^	9.83 ± 0.75 ^c^	5.00 ± 3.52 ^a^
29	74.00 ± 12.57 ^a^	48.80 ± 16.44 ^ab^	9.00 ± 0.00 ^c^	9.60 ± 0.89 ^c^	5.00 ± 2.83 ^a^
30	79.33 ± 5.72 ^a^	48.83 ± 7.03 ^ab^	8.00 ± 0.00 ^b^	8.33 ± 0.52 ^b^	5.17 ± 3.37 ^a^
31	77.33 ± 13.26 ^a^	44.00 ± 10.45 ^ab^	8.17 ± 0.41 ^b^	8.50 ± 0.55 ^b^	3.67 ± 0.52 ^a^
32	81.17 ± 7.41 ^a^	50.17 ± 10.46 ^ab^	9.33 ± 0.52 ^c^	10.00 ± 0.63 ^c^	5.00 ± 2.97 ^a^
33	73.33 ± 7.97 ^a^	64.33 ± 13.88 ^b^	7.00 ± 0.00 ^a^	7.00 ± 0.00 ^a^	2.67 ± 1.03 ^a^
Overall	76.98 ± 10.48	49.72 ± 12.48	9.47 ± 1.99	9.81 ± 1.96	4.64 ± 2.45
*House cricket*					
23	84.00 ± 4.77 ^b^	42.17 ± 15.56 ^ab^	18.00 ± 0.00 ^d^	18.33 ± 0.52 ^d^	4.00 ± 0.00 ^ab^
25	75.50 ± 12.13 ^ab^	35.50 ± 4.76 ^a^	16.00 ± 0.00 ^c^	17.00 ± 0.00 ^c^	5.67 ± 0.52 ^bc^
27	74.00 ± 7.29 ^ab^	52.00 ± 17.69 ^ab^	15.33 ± 0.82 ^c^	16.67 ± 0.82 ^c^	5.67 ± 1.37 ^bc^
29	76.50 ± 4.93 ^ab^	35.50 ± 6.28 ^a^	15.33 ± 0.52 ^c^	16.00 ± 0.63 ^c^	6.00 ± 0.89 ^c^
30	75.50 ± 4.59 ^ab^	58.00 ± 11.47 ^ab^	11.00 ± 0.00 ^b^	11.33 ± 0.52 ^ab^	2.67 ± 1.21 ^a^
31	70.50 ± 7.59 ^a^	55.00 ± 6.98 ^ab^	10.00 ± 0.00 ^a^	10.75 ± 1.50 ^a^	4.00 ± 0.00 ^ab^
32	84.50 ± 6.75 ^b^	64.83 ± 13.41 ^b^	11.67 ± 0.52 ^b^	12.17 ± 0.41 ^b^	5.83 ± 1.47 ^bc^
33	75.83 ± 3.82 ^ab^	59.67 ± 13.28 ^b^	11.00 ± 0.00 ^b^	11.17 ± 0.41 ^ab^	2.83 ± 1.17 ^a^
Overall	77.33 ± 7.75	50.13 ± 15.60	13.70 ± 2.78	14.33 ± 2.97	4.61 ± 1.63
*p*-value					
Temp	0.008	<0.001	<0.001	<0.001	0.004
Cricket	0.944	0.798	<0.001	<0.001	0.874
Temp × Cricket	0.212	0.030	<0.001	<0.001	0.143

^abcde^ Means having different superscripts within the same column for each cricket species are significantly different (*p* < 0.05), Temp: effect of incubation temperature, Cricket: effect of cricket species.

**Table 3:** Linear regression relationship between the incubation temperature (X) and hatchability pattern (Y) of two-spotted and house crickets (Experiment I).

Item	Regression	*R* ^2^	*p*-Value
	*Two-spotted cricket*		
Overall hatching rate (%)	Y = 75.256 + 0.060X	0.000	0.899
Peak daily hatching rate (%)	Y = 28.166 + 0.750X	0.040	0.176
Onset of hatching (Day)	Y = 24.620 − 0.527X	0.784	<0.001
Day of peak daily hatching (Day)	Y = 24.395 − 0.507X	0.745	<0.001
Duration from onset to the last hatching (Day)	Y = 10.429 − 0.201X	0.075	0.062
	*House cricket*		
Overall hatching rate (%)	Y = 84.670 + 0.256X	0.012	0.465
Peak daily hatching rate (%)	Y = −18.021 + 2.379X	0.260	<0.001
Onset of hatching (Day)	Y = 35.187 − 0.750X	0.813	<0.001
Day of peak daily hatching (Day)	Y = 37.010 − 0.792X	0.791	<0.001
Duration from onset to the last hatching (Day)	Y = 7.646 − 0.106X	0.047	0.146

**Table 4:** Egg hatchability pattern of the experimental two-spotted and house crickets at different incubation temperatures and relative humidity levels (Experiment II).

	Temp: 29 °C			Temp: 30 °C			Temp: 31 °C			Temp: 32 °C			SEM	*p*-Value		
Item	Hum 65%	Hum 70%	Hum 75%	Hum 65%	Hum 70%	Hum 75%	Hum 65%	Hum 70%	Hum 75%	Hum 65%	Hum 70%	Hum 75%		Temp	Hum	Temp × Hum
	*Two-spotted cricket*															
Overall hatching rate (%)	68.75 ^abc^	75.00 ^bcd^	66.50 ^ab^	66.75 ^ab^	77.25 ^bcd^	68.25 ^abc^	68.50 ^abc^	79.75 ^d^	64.25 ^a^	68.25 ^abc^	77.50 ^cd^	65.75 ^ab^	0.90	0.970	<0.001	0.552
Peak daily hatching rate (%)	22.75 ^a^	39.50 ^abcd^	30.25 ^abc^	26.25 ^ab^	33.25 ^abc^	32.00 ^abc^	26.75 ^ab^	34.25 ^abc^	44.25 ^abcd^	62.50 ^d^	48.50 ^bcd^	52.75 ^cd^	2.12	<0.001	0.313	0.054
Onset of hatching (Day)	13.00 ^d^	11.75 ^cd^	12.00 ^cd^	11.25 ^c^	9.00 ^b^	8.50 ^b^	6.00 ^a^	6.00 ^a^	6.00 ^a^	11.25 ^c^	10.75 ^c^	11.25 ^c^	0.32	<0.001	<0.001	0.011
Day of peak daily hatching (Day)	14.00 ^e^	13.75 ^de^	13.00 ^cde^	13.75 ^de^	10.75 ^bc^	9.75 ^ab^	9.25 ^ab^	8.00 ^a^	7.75 ^a^	11.25 ^bcd^	11.00 ^bc^	11.25 ^bcd^	0.33	<0.001	<0.001	0.030
Duration from onset to the last hatching (Day)	6.00 ^c^	6.25 ^c^	4.75 ^abc^	5.75 ^bc^	5.25 ^abc^	4.00 ^abc^	4.25 ^abc^	4.50 ^abc^	2.75 ^a^	2.50 ^a^	3.00 ^ab^	3.00 ^ab^	0.24	<0.001	0.023	0.549
	*House cricket*															
Overall hatching rate (%)	79.50	79.25	62.50	65.50	72.00	77.00	66.50	77.75	76.25	74.00	75.75	71.50	1.26	0.871	0.168	0.014
Peak daily hatching rate (%)	31.75 ^a^	38.50 ^ab^	31.25 ^a^	65.50 ^c^	62.00 ^c^	65.25 ^c^	64.75 ^c^	75.50 ^c^	68.00 ^c^	63.50 ^c^	59.25 ^bc^	55.00 ^bc^	2.36	<0.001	0.478	0.548
Onset of hatching (Day)	16.50 ^c^	16.75 ^c^	16.50 ^c^	12.00 ^a^	12.00 ^a^	12.00 ^a^	13.00 ^b^	13.00 ^b^	13.00 ^b^	12.50 ^ab^	13.00 ^b^	12.75 ^ab^	0.26	<0.001	0.323	0.804
Day of peak daily hatching (Day)	17.50 ^d^	17.00 ^cd^	16.75 ^c^	12.00 ^a^	12.25 ^a^	12.25 ^a^	13.00 ^b^	13.00 ^b^	13.00 ^b^	13.00 ^b^	13.00 ^b^	13.00 ^b^	0.28	<0.001	0.507	0.062
Duration from onset to the last hatching (Day)	6.25 ^c^	5.25 ^bc^	5.00 ^bc^	1.00 ^a^	1.50 ^a^	1.75 ^a^	1.75 ^a^	2.00 ^a^	2.25 ^a^	2.50 ^a^	3.00 ^ab^	5.00 ^bc^	0.28	<0.001	0.147	0.027

^abcde^ Means having different superscripts within the same row are significantly different (*p* < 0.05); Temp: incubation temperature/effect of incubation temperature; Hum: relative humidity/effect of relative humidity.

## Data Availability

The original contributions presented in the study are included in the article. Further inquiries can be directed to the corresponding author.
